# Preparation and Application of pH Self-Controlled Slow-Release Sensor

**DOI:** 10.3390/gels12040308

**Published:** 2026-04-03

**Authors:** Lan Yang, Qian-Yu Yuan, Ching-Wen Lou, Jia-Horng Lin

**Affiliations:** 1 School of Textile Science and Engineering, Tiangong University, Tianjin 300387, China; lanyang202210@163.com (L.Y.);; 2 Department of Bioinformatics and Medical Engineering, Asia University, Taichung City 413305, Taiwan; 3 Ministry of Education Key Laboratory for Advanced Textile Composite Materials, Tiangong University, Tianjin 300387, China; 4 Advanced Medical Care and Protection Technology Research Center, Department of Fiber and Composite Materials, Feng Chia University, Taichung City 407102, Taiwan

**Keywords:** Eudragit L100, pH sensor, food packaging, antibacterial, antibacterial, antioxidant

## Abstract

Current smart packaging systems exhibit uneven release of active ingredients (rapid in the early stage and slow in the later stage), resulting in insufficient antibacterial and antioxidant properties. This study developed a pH-autonomous controlled-release sensor using Eudragit L100 and citrate as the matrix, with eugenol as the active component, and constructed a sandwich structure via electrospinning. The sensor can automatically release eugenol as needed in response to pH changes during shrimp storage, while simultaneously enabling visual monitoring of spoilage status. This innovation effectively extends the shelf life of fresh shrimp and provides a novel solution for the on-demand release of active ingredients in food preservation.

## 1. Introduction

Novel smart packaging materials are designed to extend the shelf life of food by incorporating natural active ingredients. However, many existing packaging materials exhibit rapid initial release of active components followed by gradual deceleration during storage [1,2], resulting in low overall antibacterial efficacy and antioxidant capacity. Ideal packaging materials should demonstrate controlled release of active ingredients under non-microbial conditions, while rapidly releasing them to inhibit microbial growth when exposed to microbial threats. Consequently, regulating active-ingredient release to prolong food shelf life has become a key research focus [3,4]. Controlled-release technology, an emerging approach based on microbial growth kinetics, enables precise release of active components as needed [5,6]. This technology typically utilizes external stimuli (e.g., temperature, light) or internal environmental factors (e.g., pH, humidity) to alter the structure or properties of packaging materials, thereby modulating release rates. Although various materials have been developed for controlled release in recent decades, research on packaging for fresh shrimp preservation remains relatively limited [7,8].

Among various responsive strategies, pH-responsive systems have garnered significant attention due to their high correlation with food spoilage processes induced by microbial metabolic activities. Recent studies have made significant progress in pH-responsive smart packaging. Noori et al. incorporated cobalt-based metal–organic framework nanoparticles loaded with berberine into polyvinyl alcohol/chitosan nanofiber membranes, developing a multifunctional packaging for red meat preservation that achieves dual functions of freshness monitoring and antibacterial activity [9]. Jiang et al. prepared a pH-responsive smart membrane based on soybean lipophilic protein/hydroxypropyl methylcellulose for salmon freshness monitoring, where color changes are highly correlated with volatile alkaline substances produced during fish spoilage [10]. Additionally, pH-responsive smart membranes containing anthocyanin cations have been widely applied in freshness monitoring of various foods, reflecting food quality status in real time through visually discernible color changes [11]. However, existing pH-responsive antibacterial packaging primarily achieves release through pH-triggered membrane swelling or bond cleavage, such as Schiff base hydrolysis under acidic conditions releasing antibacterial agents, which enables a mode transition from defense to attack [12], or MIL-101 (Fe) loaded with allantoin disintegrating in the acidic environment generated by fruit spoilage to achieve responsive release [13]; both rely on structural disintegration or chemical bond cleavage of the packaging material under specific pH conditions to release active ingredients, belonging to passively triggered release. Additionally, there are other examples such as Pickering emulsion films, which achieve pH-responsive release, but their release kinetics may not fully match the microbial growth curves (especially the logarithmic growth phase) of specific foods (e.g., fresh shrimp), potentially leading to issues of response lag or release mismatch.

**Figure 1:**
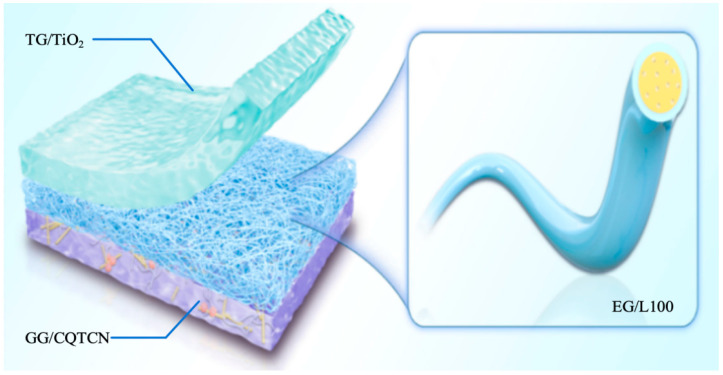
Schematic diagram of pH auto-controlled sustained-release sensor.

Distinguished from the traditional passive response release, this study developed a pH-controlled sustained-release sensor to monitor real-time internal pH fluctuations in fresh shrimp during storage and regulate the release rate of active ingredients (Figure 1). Eudragit L100 (L100), a polymer synthesized from methyl methacrylate and ethyl acrylate copolymers, enables controlled release of active components under preset conditions by incorporating weak acids or alkaline salts, meeting specific preservation requirements for various foods. Using eugenol as the active ingredient and L100 with trace citrate as the polymer matrix, a sandwich structure was fabricated via electrospinning technology. This structure not only automatically releases eugenol in response to internal pH changes to prolong food preservation but also provides visual monitoring of shrimp spoilage. Innovation offers a novel solution for on-demand release of active ingredients [14] during food preservation.

## 2. Results and Discussion

### 2.1. Microstructural Characterization of pH-Controlled Sustained-Release Layers

#### 2.1.1. Scanning Electron Microscopy (SEM) Observation

**Figure 2:**
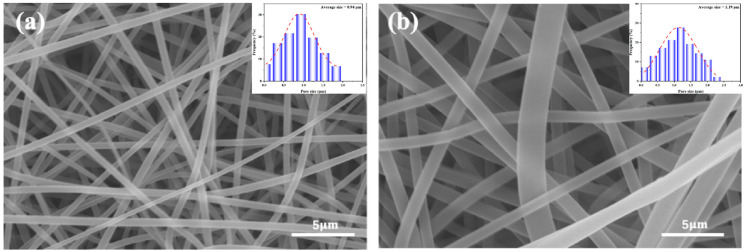
(**a**) SEM image of the upper surfaces of EG/L100 and (**b**) SEM image of the lower surfaces of EG/L100 nanosheets.

Figure 2 presents SEM images of the upper and lower surfaces of the EG/L100 nanosheet intercalation. The images demonstrate that the prepared EG/L100 nanosheet intercalation exhibits a continuous and uniform distribution across both surfaces (upper surface refers to the surface facing the exterior of the package, lower surface refers to the surface facing the interior of the food) (Figure 2a,b). The fibers are smooth and free of obvious defects, while the nanofiber membrane surface features abundant pores, facilitating rapid water transport and diffusion within the membrane. The results indicate that the upper-layer membrane has smaller and more concentrated pores with an average pore size of approximately 0.18 μm, whereas the lower-layer membrane has slightly larger pores and a looser structure with an average pore size of about 1.50 μm. This gradient pore structure creates a significant capillary pressure difference in EG/L100, optimizing the water transport pathway. During the spoilage process of fresh shrimp, exudates and volatile alkaline substances can be rapidly absorbed and directed to the intermediate layer. When the pH approaches or exceeds its critical value, the intermediate layer undergoes chain segment relaxation, allowing the embedded EG to gradually diffuse to the surface of the shrimp. In summary, the EG/L100 nanosheet intercalation ensures stable unidirectional water transport performance through its differentiated pore distribution, enabling rapid sensing of microenvironmental changes on food surfaces and pH-triggered controlled release. This design combines the advantages of efficient mass transfer, directional transport, and pH-sensitive controlled release, providing robust structural and mechanistic support for intelligent antibacterial food packaging.

#### 2.1.2. Infrared Spectroscopy (FTIR)

**Figure 3:**
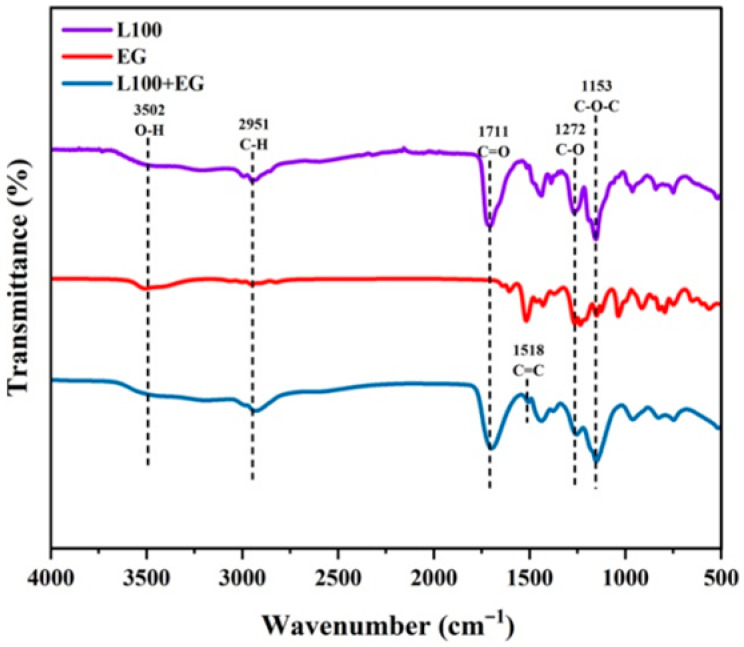
pH-controlled release nano-intercalation infrared spectroscopy.

Figure 3 presents the pH-controlled release infrared spectra of nano-intercalated materials. The spectra demonstrate significant intermolecular interactions between L100 and EG, establishing the structural foundation for subsequent pH-controlled release. In L100’s spectrum, the 3502 cm^−1^ peak corresponds to the hydroxyl O–H stretching vibration [15,16], while the 2951 cm^−1^ peak indicates the C–H asymmetric stretching vibration [17]. The 1711 cm^−1^ peak represents the ester carbonyl C=O stretching vibration [18,19], with the 1272 cm^−1^ and 1153 cm^−1^ peaks corresponding to C–O and C–O–C stretching vibrations, respectively [15]. For EG, the 3510 cm^−1^ peak shows the phenolic hydroxyl O–H stretching vibration, and the 2955 cm^−1^ peak indicates the C–H asymmetric stretching vibration. The 1608 cm^−1^ and 1513 cm^−1^ peaks correspond to aromatic ring C=C backbone vibrations [20], while the 1235 cm^−1^ and 1034 cm^−1^ peaks represent C–O and C–O–C stretching vibrations [21]. After the addition of eugenol, the 3502 cm^−1^ O–H stretching vibration in the L100/EG composite system shifts toward lower wavenumbers with slight broadening, indicating hydrogen bonding interactions between L100 and eugenol that transform eugenol from a free state to a polymer-network-bound conformation [22]. Additionally, the C=C stretching vibration peak at approximately 1518 cm^−1^, attributed to the eugenol aromatic ring backbone, overlaps with partial vibration bands of L100, further confirming successful encapsulation of eugenol within the L100 matrix rather than simple surface adsorption. The hydrogen bonding interaction between Eudragit-type acrylic copolymers and phenolic active substances can significantly enhance the stability of volatile phenols and slow down their diffusion rate, making their release behavior more dependent on the ionization and swelling state of the carrier, thereby forming a pH-triggered sustained-release characteristic.

### 2.2. X-Ray Diffraction Energy Spectroscopy (XRD)

**Figure 4:**
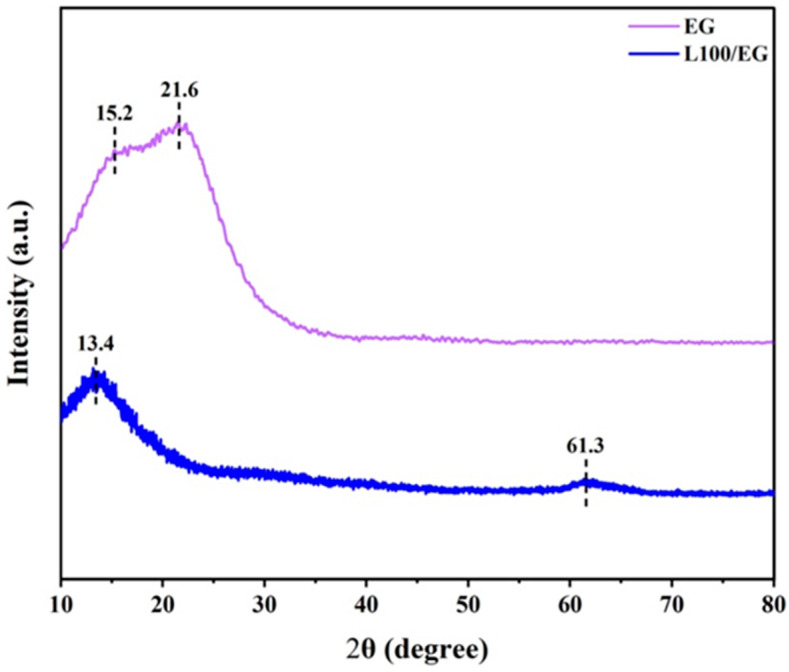
X-ray diffraction energy spectrum of pH-controlled release nano-intercalated materials.

As shown in Figure 4, the XRD results effectively reveal the loading state of EG in L100 electrospun fibers and its regulatory effect on the matrix crystallization behavior. Pure EG exhibits sharp and distinct diffraction peaks at 2θ ≈ 15.2° and 21.6°, demonstrating typical small-molecule organic crystal characteristics [23]. This indicates that eugenol molecules are arranged in a long-range ordered manner with a regular lattice structure, suggesting a predominantly crystalline system. However, in the diffraction spectra of L100/EG composite fiber membranes, all characteristic crystalline peaks attributable to EG completely disappear, with no residual signals from EG’s own crystalline phase observed. This indicates that during the blending and electrospinning process with L100, EG’s original crystalline structure is completely disrupted, and it no longer precipitates as independent crystals. Simultaneously, new diffraction peaks appear at 2θ ≈ 13.4° and 61.3° in the L100/EG spectra [24]. This change primarily occurs because EG molecules can interact with polar groups on L100 chains through hydrogen bonding or van der Waals forces, weakening interchain interactions and making some chain segments more flexible. This induces the polymer to form new locally ordered arrangements, reflected in the shifts of diffraction peak positions and morphologies. EG molecules are uniformly dispersed in an amorphous state between L100 chain segments, disrupting the continuity of EG’s own lattice structure. This transforms the system from simple physical mixing into a single composite phase where L100 forms a continuous phase and EG is embedded and dispersed in an amorphous state. If only surface adsorption or rough mixing occurred, the characteristic peaks at 15.2° and 21.6° would still overlap and not completely disappear. Therefore, the energy spectrum demonstrates that EG has been successfully incorporated into the L100 electrospun fibers and uniformly loaded in an amorphous form within the matrix network, providing a favorable structural foundation for subsequent pH-triggered diffusion and sustained release.

### 2.3. Mechanical Properties Analysis

**Figure 5:**
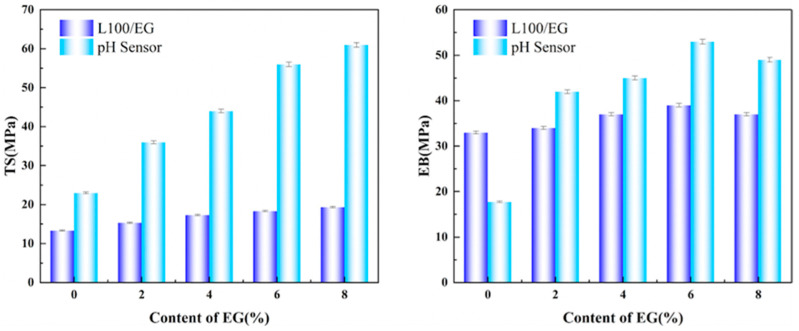
Mechanical properties analysis of EG/L100 nanosheet intercalation.

As shown in Figure 5, the TS (tensile strength) of L100 nanosheet intercalation without eugenol was approximately 13.58 MPa, with an elongation at break (EB) of about 32.16%. This exhibits a typical curve pattern of elastic stretching followed by yield elongation, indicating that the pure L100 electrospun fiber membrane already possesses good load-bearing capacity and ductility. When the eugenol content increased from 2% to 4%, the TS and EB showed only slight fluctuations, suggesting that moderate eugenol acts as an internal plasticizer and chain lubricant in L100. By partially weakening the strong intermolecular forces and interchain entanglements of L100, it facilitates reversible orientation and slip of polymer chains, thereby enhancing toughness without significant reduction in strength. When the eugenol content further increased to 6% and 8%, the TS curve peak experienced more pronounced decline, while the EB initially showed a slight increase before gradually decreasing. This is attributed to the extensive insertion of eugenol between L100 chain segments, which significantly weakens hydrogen bonds and van der Waals forces, resulting in a looser network structure and reduced load-bearing capacity. Moderate amounts of EG form hydrogen bond networks with L100 carboxyl/hydroxyl groups, maintaining the intermediate layer’s continuity, density, and tensile strength in the dry state. In the sandwich structure, EG can stably exist as a functional core layer. When food spoilage causes localized pH elevation, the ionization and swelling of the intermediate L100 layer disrupt hydrogen bonds, releasing eugenol from interchain voids and inhibiting the degree of spoilage. In conclusion, the effects of different eugenol concentrations on mechanical properties are closely related to their distribution and interactions within the polymer network. Excessive addition may compromise mechanical stability, whereas a 4% EG content significantly enhances flexibility while maintaining sufficient membrane strength for packaging requirements. This approach also provides adequate deformation space for subsequent pH-triggered swelling and chain segment rearrangement.

### 2.4. Analysis of Water Contact Angle

**Figure 6:**
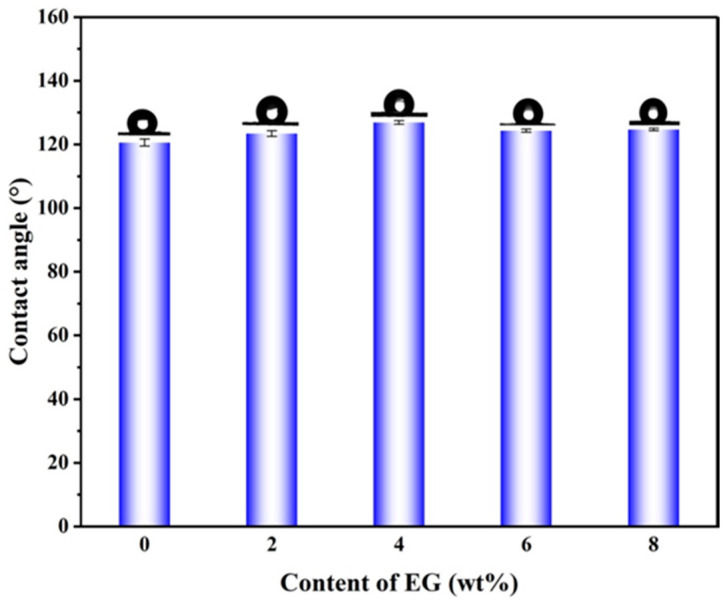
Water contact angle of EG/L100 nanosheet intercalation.

Figure 6 presents the water contact angle (WCA) analysis of EG/L100 nanosheet intercalation. The L100/EG nanosheet intercalation maintains an overall hydrophobic barrier while enabling precise control of surface wettability and pH-triggered behavior through adjustment of eugenol content. The pure L100 nanofilm exhibits a WCA of 120.57°, demonstrating typical hydrophobic characteristics, which is closely related to the non-ionic state of L100 at pH values below its pKa and the dominance of hydrophobic groups in interface properties. As eugenol content increases to 6%, the WCA rises to 126.87°, indicating enrichment of aromatic ring structures and hydrophobic side chains at the interface, further reducing surface free energy and enhancing water repulsion. When EG content increases to 8%, the WCA slightly decreases to 124.73°, primarily attributed to localized enrichment of excess eugenol on the fiber surface, forming a soft interface layer more easily wetted by water molecules, thereby partially weakening hydrophobicity. Overall, when EG content ranges from 0 to 6%, the nanofilm surface consistently maintains a WCA above 120°. This not only prevents free water infiltration during initial storage and avoids premature swelling of packaging materials but also, through the pore size gradient of the sandwich structure, enables unidirectional moisture transport via inter-fiber channels rather than overall wetting. Fresh food exhibits lower pH values, with L100 segments remaining non-ionic. The hydrophobicity of the nanofilm surface hinders solution spreading, while the L100/EG layer remains dense and stable, releasing only minimal surface eugenol to exert fundamental antibacterial and antioxidant effects. As the pH rises, the carboxyl group of L100 undergoes progressive deprotonation, causing segment swelling and hydrophilicity enhancement. This facilitates water infiltration into the fiber interior, thereby triggering sustained eugenol release. The results demonstrate the synergistic advantages of this pH-sensing membrane in interface design and intelligent controlled release.

### 2.5. Determination of Loading Rate (LC), Encapsulation Efficiency (EE), and Porosity of pH-Controlled Release Fiber Membranes

**Figure 7:**
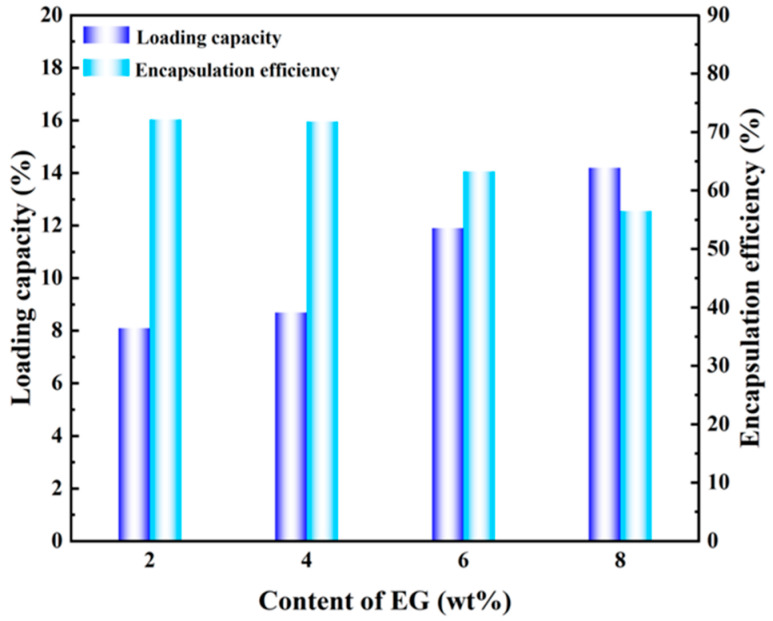
EG/L100 nanoscale intercalation loading ratio (LC) and encapsulation efficiency (EE).

Figure 7 presents the loading capacity (LC) and encapsulation efficiency (EE) of EG/L100 nanoscale intercalated membranes. The test results of pH-controlled release fiber membranes demonstrated that the addition of different concentrations of eugenol significantly influenced drug loading capacity and pore structure regulation, which were closely related to subsequent pH-triggered release behavior. Using the unloaded L100 membrane as a control, both LC and EE were 0, indicating that the fibers exhibited hydrophilic properties and served only as structural and mass transfer carriers. When eugenol concentrations reached 2% and 4%, LC increased while EE showed minimal variation, consistent with a BET specific surface area of 7.22 m^2^/g and BJH total pore volume of 0.014 cm^3^/g. This suggests that eugenol primarily existed in embedded fiber structures and dispersed within the porous network, achieving high loading efficiency while effectively preventing surface EG aggregation or phase separation. The literature also confirms that this encapsulation level did not adversely affect the food’s taste, elasticity, or flavor. As eugenol concentration further increased to 6% and 8%, theoretical LC and EE should have continued to rise, but measured EE showed a slight decrease, with pore volume and porosity exhibiting an initial increase followed by a slight decline. This phenomenon may be attributed to eugenol leakage or surface enrichment due to exceeding the effective binding and encapsulation capacity of the L100 network. Therefore, a moderate loading concentration of 4% EG (LC = 8.70%, EE = 71.80%) was optimal, enabling the fiber membrane to maintain strong drug loading and encapsulation capabilities while preserving an appropriate porous structure and mass transfer channels.

### 2.6. Analysis of Antioxidant and Antibacterial Properties of pH-Controlled Release Fiber Membranes

**Figure 8:**
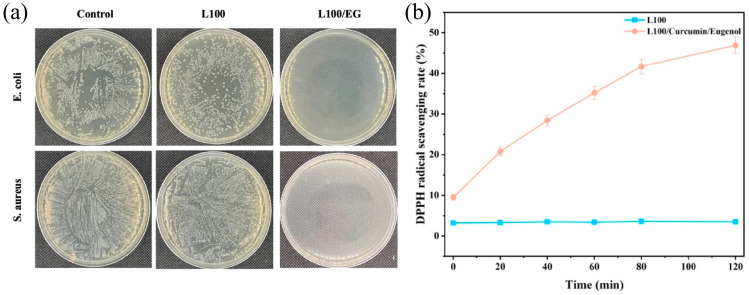
EG/L100 nanoscale intercalation (**a**) antibacterial rate and (**b**) antioxidant rate.

As shown in Figure 8a, the pH self-regulating sustained-release sensor exhibited significant broad-spectrum antibacterial properties after the introduction of eugenol. The colony counts of E. coli and S. aureus in the L100 (0% EG) nanofilm showed no significant changes, indicating that L100 itself had minimal antibacterial activity. In the L100/EG group, almost no visible colonies were observed, demonstrating a typical comprehensive antibacterial coverage effect, which confirmed the successful release of eugenol in the carrier and its effective antibacterial capability. Eugenol disrupts bacterial cell membrane structure, inhibits the activity of key metabolic enzymes, and disrupts energy metabolism, ultimately leading to cell death. In the late stage of shrimp spoilage, eugenol gradually ionizes and swells with increasing pH, thereby achieving sustained-release control and targeted bacterial suppression. This effectively inhibits the growth of spoilage-causing bacteria, significantly extends shelf life, and prevents excessive exposure of active compounds. Figure 8b shows the DPPH scavenging experiment. The free radical scavenging rate of pure L100 nanofilm remained consistently at approximately 3.5% ± 0.4% and showed almost no change over time, indicating that the carrier polymer itself did not participate in the free radical scavenging process. In contrast, the scavenging rate of L100 nanofilm loaded with eugenol increased continuously from an initial 9.5% ± 0.7% to 46.9% ± 2.0% (120 min), demonstrating a clear time-dependent trend. The phenolic hydroxyl groups on eugenol molecules can act as hydrogen donors, reacting with free radicals such as DPPH to form stable phenol oxygen radicals, thereby terminating free radical chain reactions. Leveraging the pH-responsive controlled-release properties of L100 and the unidirectional water-guiding effect of its sandwich structure, the localized pH elevation and exudate accumulation caused by shrimp spoilage trigger L100’s swelling and ionization. This accelerates the release of conjugated eugenol, enabling targeted enhancement of antioxidant activity during the spoilage phase when lipid oxidation is most likely to occur. Thus, the L100/EG nano-intercalation achieves intelligent coupling of antioxidant activity with pH fluctuations and moisture migration, effectively delaying shrimp spoilage. Outperforming conventional antioxidants in standard packaging, this material demonstrates comprehensive advantages as a pH-sensitive freshness-preserving packaging material.

### 2.7. Determination of Cytotoxicity in pH-Controlled Release Fibrous Membranes

**Figure 9:**
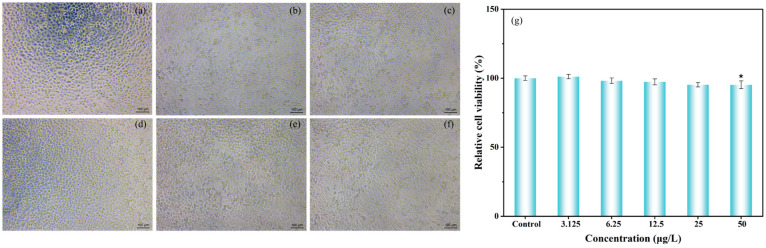
Cell survival rate of white light at different concentrations: (**a**) control group, (**b**) concentration 3.125, (**c**) concentration 6.25, (**d**) concentration 12.5, (**e**) concentration 25, (**f**) concentration 50. (**g**) Cell survival rate of double-layer membrane extract at different concentrations.

Figure 9 presents the white-light viability curves of cells at different concentrations. The L100/EG nano-intercalation exhibits excellent biocompatibility in toxicology, meeting safety requirements for direct food contact and partial structural dissolution. Cytotoxicity results demonstrate that the relative survival rate of mouse fibroblasts remains consistently above 85% within the concentration range of 3.125–12.5 μg/mL. Even at 50 μg/mL, cell viability remains stable above 80%, indicating minimal biological interference of the fibrous membrane dissolution product on mammalian cells, which is within an acceptable safety range. L100 acrylic copolymer, widely used in oral sustained-release formulations, has been proven to exhibit good gastrointestinal compatibility. It undergoes controlled dissolution under near-neutral or slightly alkaline conditions with extremely low intrinsic toxicity. Eugenol, a naturally derived aromatic phenol commonly used in spices and natural preservatives, demonstrates high safety for both cells and humans. In this study, the core–shell structure encapsulates EG within L100, avoiding excessive EG exposure and reducing irritancy. Integrating the measured cytotoxicity results with material composition characteristics, the L100/EG nano-intercalation achieves high-efficiency antibacterial and pH-controlled release while maintaining excellent cellular compatibility, providing robust support for its application as a smart active packaging material for direct food contact.

### 2.8. Determination of Water Vapor Transmission Rate of pH Sensor

**Figure 10:**
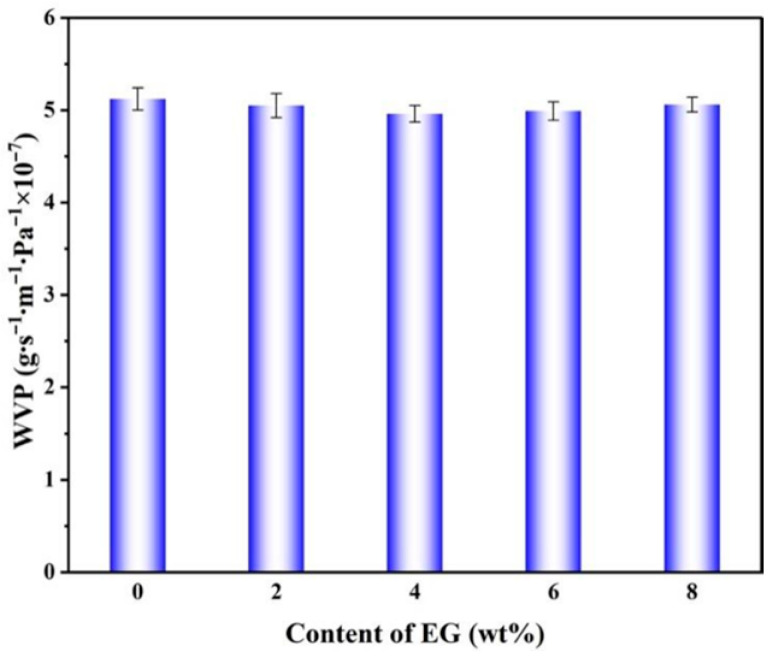
Vapor permeability of nano-layered water with different EG addition amounts.

Figure 10 presents the vapor permeability (WVP) curves of nanoscale intercalated membranes with varying EG content. The L100 system exhibited minimal WVP fluctuations, which aligns well with its inherent high porosity and strong chain segment relaxation characteristics. This suggests that eugenol, as a hydrophobic small-molecule additive, exerts a “fine-tuning” rather than “fundamental alteration” effect on water vapor transport. Under experimental conditions, L100 demonstrates moderate hydrophobicity, while its high porosity and substantial free volume fraction enable efficient vapor diffusion through the membrane, resulting in relatively high WVP values for pure L100 membranes. When a small amount of eugenol (2–4%) is introduced, EG acts as a hydrophobic filler. During fiber formation, it partially fills interchain free volumes and local micropores in L100, while non-covalent interactions (e.g., hydrogen bonds and van der Waals forces) between EG and L100 carboxyl/ester groups restrict excessive chain relaxation. This process slightly densifies the fiber walls and reduces equivalent permeable pores, leading to a modest decrease in WVP from 5.12 × 10^−7^ to approximately 4.96 × 10^−7^ g/(m·s·Pa). When EG content increases further to 6–8%, excess small molecules begin to accumulate locally or form phase separations. This may induce slight reductions in fiber diameter and increases in pore size, or create more permeable regions at fiber intersections, partially reopening effective vapor channels. Additionally, excessive EG enhances L100’s plasticizing effect, increasing chain segment mobility and dynamic free volume, which causes a slight rebound in WVP but ultimately maintains levels comparable to pure membranes. This moderately adjustable rather than drastically reduced water vapor permeability (WVP) property is particularly crucial for active food packaging. On one hand, it maintains sufficient water vapor permeability in the L100/EG fiber membrane, allowing humidity and pH variations in the food surface microenvironment to be effectively transferred into the fiber interior. This triggers structural relaxation of L100 when pH increases, thereby driving the directional release of eugenol from its amorphous dispersed state to the interface. Simultaneously, WVP remains significantly unchanged, ensuring the L100/EG fiber membrane retains its fundamental moisture barrier capability. This prevents excessive water vapor penetration from causing rapid membrane collapse or mechanical property degradation, providing a favorable structural foundation and mass transfer condition for the long-term antibacterial preservation of fresh shrimp.

### 2.9. Analysis of the Release Mechanism of pH-Controlled Sustained-Release Layer

**Figure 11:**
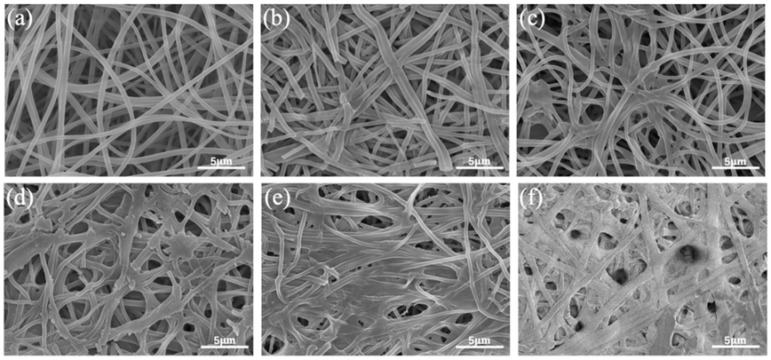
SEM images of EG/L100 nanosheets intercalated at different pH levels: (**a**) pH 5.25, (**b**) pH 5.50, (**c**) pH 5.75, (**d**) pH 6.00, (**e**) pH 6.25, (**f**) pH 6.50.

**Figure 12:**
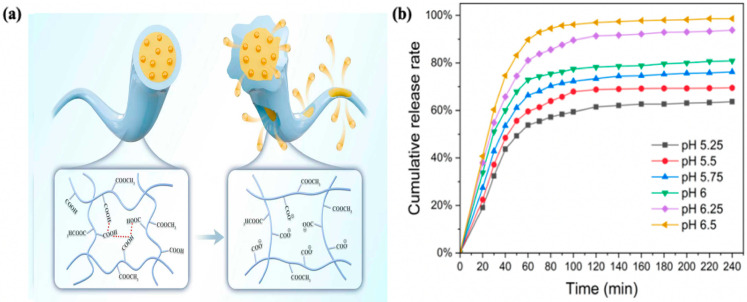
EG/L100 Nanointercalation (**a**) Mechanism of action, (**b**) Cumulative release rate in different pH environments.

The EG/L100 nanoscale intercalation exhibited typical pH-controlled release characteristics under different pH conditions, demonstrating the L100 carrier’s significant pH-responsive regulation capability for clove essential oil. The release curve demonstrates that during the initial 60 min, when the external pH is below the pKa of L100, the carboxyl groups exist in a non-ionized form, with intermolecular hydrogen bonding and hydrophobic interactions dominating. The chain segments tightly entangle, and inter-fiber adhesion increases, forming a relatively dense encapsulated structure (as shown in Figure 11a–c, where the fibers are more compactly arranged). At this stage, the clove essential oil primarily relies on its own concentration gradient for slow diffusion, exhibiting low-rate release. As the pH gradually increases from 5.25, the time required for the system to reach equilibrium is significantly shortened, and the cumulative release rate of the essential oil rises sequentially from 61.9% to 97.2%. This indicates that when the pH exceeds the pKa of L100, the carboxyl groups on the L100 chains undergo deprotonation to form –COO^−^, leading to increased electrostatic repulsion between chain segments. The polymer transitions from agglomerative contraction to swelling and extension, with the fiber structure exhibiting characteristics such as enlarged pores and weakened cross-linking points (as shown in Figure 11d–f, where the fibers gradually loosen, fracture, and collapse into porous structures). This not only provides a more unobstructed transmission channel for the diffusion of the essential oil but also the charge repulsion itself serves as an additional driving force for the outward migration of the core layer essential oil, thereby achieving rapid and sufficient release under high-pH conditions. Finally, the system enters a slow-release phase controlled by diffusion and polymer chain segment movement, gradually reaching a plateau equilibrium. The cumulative release rates of the essential oil are 61.9%, 68.3%, 72.1%, 76.6%, 91.4% and 97.2%, respectively. The differences in release rates primarily stem from structural changes in the matrix and the concentration gradient of the active component [25], with the underlying mechanism illustrated in Figure 12a. The EG/L100 nanoscale intercalation achieves adaptive regulation of inhibition of release at low pH and accelerated release at high pH, truly realizing the on-demand antibacterial strategy of the more severe the spoilage, the more thorough the release. This material combines the advantages of high-efficiency preservation with intelligent indication.

**Figure 13:**
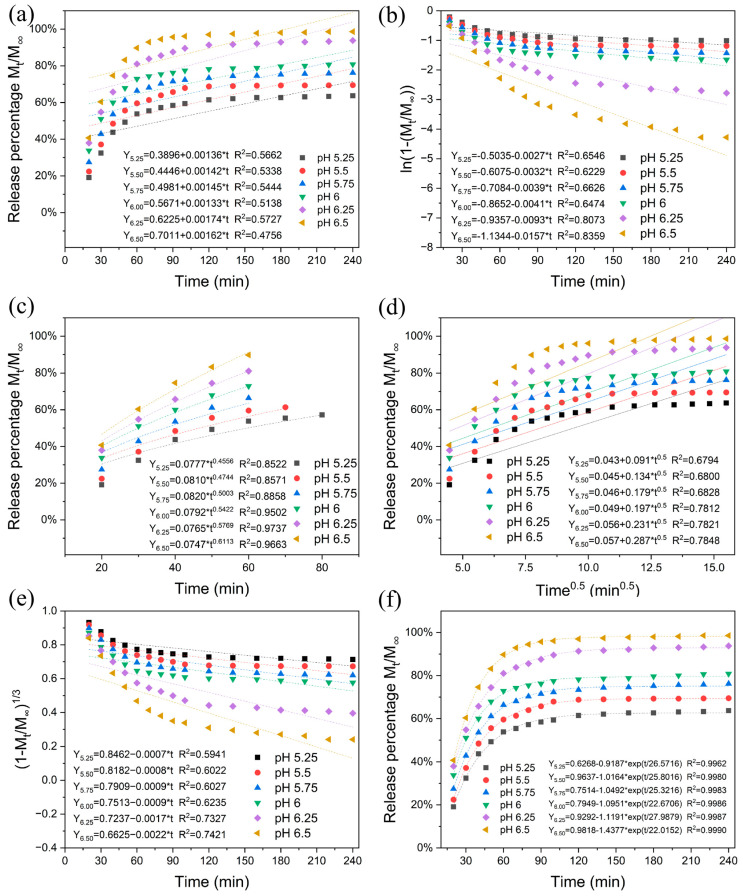
Six release kinetics models of EG: (**a**) zero-order, (**b**) first-order, (**c**) Ritger–Peppas, (**d**) Higuchi, (**e**) Hixson–Crowell, (**f**) two-way kinetics.

Figure 13 presents the fitting analysis of release behavior using six typical release kinetics models. The cumulative release rate was converted into release percentage (Mt/M∞) as the vertical axis. Comparative analysis of correlation coefficients R^2^ among models revealed that the biphasic kinetics model demonstrated the highest fit (R^2^ = 0.9962–0.9990), most accurately describing the release process of this system. The model results indicate that when pH > 6, the shell polymer matrix of L100@EG fiber membrane rapidly transitions from solid to liquid phase, thereby accelerating essential oil release. The Ritger–Peppas model also exhibited high correlation (R^2^ = 0.8522–0.9663), further confirming the regularity of release behavior. According to the definition of release index *n* in this model [26], *n* < 0.50 indicates Fickian diffusion, *n* > 0.50 belongs to non-Fickian (dissolution diffusion) mechanism, and *n* ≥ 1.0 represents zero-order release. The study results show that with increasing pH, the *n* value rises from 0.4556 to 0.6113, indicating a proportional relationship between release mechanism and pH. The fitting results of first-order kinetics models and the Hixson–Crowell model also effectively explain the pH-controlled release characteristics of fiber membranes. In summary, the release kinetics analysis results corroborate the previously proposed polymer structure response mechanism, further supporting the conclusion that the CQTCN/TiO_2_/L100@EG sensor possesses pH-triggered and controlled-release functions.

### 2.10. Application of pH Sensor in Freshness Monitoring of Fresh Shrimp

#### 2.10.1. Study on the Freshness Indication Effect of pH Sensor

**Figure 14:**
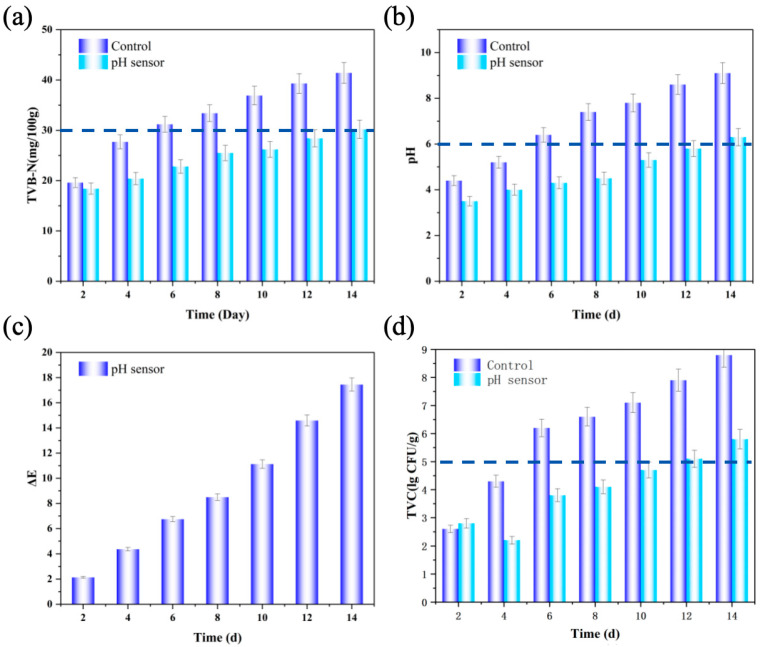
(**a**) Changes in TVB-N at 14 days between the control group and the experimental group. (**b**) Changes in pH at 14 days between the control group and the experimental group. (**c**) Color difference values at 14 days in the experimental group. (**d**) Changes in TVC at 14 days between the control group and the experimental group.

**Figure 15:**
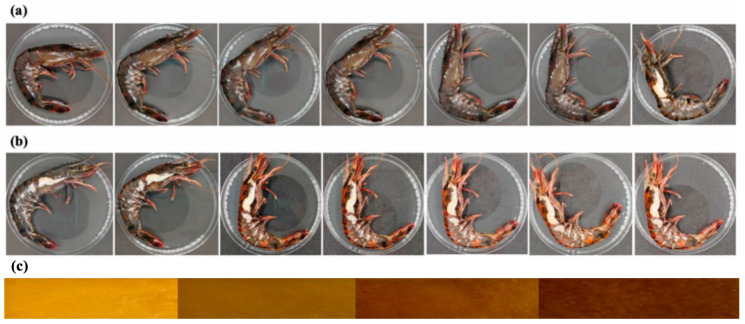
Freshness changes of tiger shrimp at −4 °C for 14 days: (**a**) experimental group, (**b**) control group. (**c**) Color-changing physical image of the sensing layer (pH < 6; pH = 7; pH 8~11; pH 12~13).

The pH sensor was applied to the active packaging of fresh shrimp, and the preservation effect is shown in Figure 14. Under the condition of −4 °C, the freshness changes of tiger prawns after being placed for 14 days, as shown in Figure 15a experimental group, Figure 15b control group. Figure 15c Color change images of the sensing layer (pH < 6; pH = 7; pH 8–11; pH 12–13). The differences in various indicators between the control group and the experimental group were small in the first two days. From the fourth day, the TVB-N value of the control group rose to 27.7 mg/100 g, which exceeded the limit for fresh shrimp stipulated in the national standard GB 2707-2016 [27] (TVB-N < 30 mg/100 g), indicating that the shrimp meat was in a subfresh state. On the fifth day, it had already exceeded the fresh shrimp freshness limit. While the TVB-N value of the experimental group on the fourth day was 20.4 mg/100 g, the shrimp meat was still within the edible range. This indicates that the entire shrimp sample was effectively protected rather than just a localized area. If only the region directly below the release point was protected, microbial proliferation in the uncovered area would lead to a rapid increase in both total bacterial count and TVB-N value, which contradicts the observed results. On the tenth day, the TVB-N value was 26.2 mg/100 g, still meeting the freshness requirements. When the fresh shrimp was stored for the 14th day, the TVB-N value rose to 30.2 mg/100 g, indicating that the shrimp meat had gone bad. Figure 14d shows the change in the total number of colonies. On the fourth day, the surface colony count of the experimental group was significantly lower than that of the control group (4.3 lgCFU/g), because the sensor released a large amount of eugenol, which significantly inhibited the growth of microorganisms. On the 14th day, the colony count of the experimental group reached 5.8 lgCFU/g, while that of the control group had reached 8.8 lgCFU/g. Therefore, under the storage condition of −4 °C, the pH sensor can extend the shelf life of fresh shrimp products by approximately 9 days. Observing the ΔE change of the indicator film (when ΔE > 5, it is a visible color change), the indicator film of the experimental group had ΔE < 5 (light yellow) in the first 5 days (fresh period), and ΔE > 5 (orange yellow) in days 5 to 9 (subfresh period), proving that the controlled release of active ingredients effectively enhanced the preservation effect. On the 14th day, the indicator film turned orange. Therefore, it was confirmed that this sensor can reflect the freshness of fresh shrimp in real time through color changes, enabling synchronous monitoring and regulation during the storage process. Due to its moderate volatility, eugenol can migrate through vapor-phase diffusion in the packaging headspace. Upon release initiation, eugenol first releases from the core–shell fibers, traversing the porous network of the underlying hydrogel to reach the shrimp meat surface. Simultaneously, a portion of eugenol volatilizes into the packaging headspace, achieving uniform distribution throughout the packaging space via gas-phase diffusion. When the system reaches dynamic equilibrium, the eugenol vapor concentration in the headspace becomes homogeneous, ensuring all shrimp meat sections are exposed to a similar concentration of antimicrobial environment. This experiment demonstrates that the sensor does not passively respond after spoilage occurs, but initiates low-dose antimicrobial release during the early stages of spoilage risk. As spoilage risk increases (with continued pH elevation), the release intensity progressively intensifies, forming an intelligent preservation mechanism of “risk perception–gradient response–sustained defense.” This design ensures precise alignment between the antimicrobial release timing and the critical microbial growth window (late lag phase to early logarithmic phase), effectively inhibiting microbial proliferation before spoilage occurs, rather than providing remedial measures after spoilage.

#### 2.10.2. Cost Analysis of pH Sensor in Seafood Product Packaging

All raw materials used in this research are food-grade or pharmaceutical-grade components, characterized by low toxicity, controllable costs, and suitability for large-scale production. TG and GG are commonly used food-grade polysaccharide raw materials, widely applied in dairy products, candies, and frozen foods. They are safe and non-toxic within the permitted regulatory addition limits, with market prices typically ranging from tens of RMB/kg, making them low-cost raw materials. PVDF, depending on the brand, has domestic prices as low as approximately 40 RMB/kg. Eudragit L100, copolymerized from methyl methacrylate and ethyl acrylate, has been long used in oral sustained-release formulations as an enteric coating. Recognized by pharmacopeias and regulatory agencies as a safe pharmaceutical excipient, its unit price is higher than that of ordinary polysaccharides, but its usage in films is minimal, resulting in a limited overall cost proportion. The main raw materials of CQTCN are microcrystalline cellulose and curcumin. MCC is priced at approximately 16 RMB/kg, while curcumin and clove bud extract are derived from common natural aromatic plants, proven to possess excellent safety and antioxidant/antibacterial activities. They are widely used in condiments and health foods, with moderate raw material costs and minimal usage per unit membrane area. According to the national standard GB 2760-2014 [28], TiO_2_ and CTAC can be used as food contact materials or food additives within specified limits and applications. TiO_2_ has a unit price of approximately 12 RMB/kg, and CTAC, a common quaternary ammonium salt surfactant, is significantly cheaper than functional active ingredients. Based on current domestic market procurement price estimates (material costs of the film are shown in Table 1), the material cost for producing a 20 mm square composite film while maintaining active-ingredient payload is approximately 1.5–2 yuan. This cost is significantly lower than most high-end multi-layer co-extruded or nano-inorganic-filled packaging materials, offering advantages in non-toxicity, safety, and cost-effectiveness. The material is suitable for further scale-up production and industrial evaluation.

#### 2.10.3. Design and Application of pH Sensor in Seafood Product Packaging

This study integrates cutting-edge material science with market-oriented applications, developing a pH-sensing freshness-preserving packaging for seafood like black tiger prawns. The innovative design features on-demand controlled release and visual alerts. The packaging’s front displays a circular smart window matching the internal sensing membrane, equipped with a freshness indicator color panel (transitioning from light yellow to orange–yellow and then to orange–red) that visually tracks product quality changes from fresh to near-spoilage. During initial storage, when ambient pH is low, L100 remains in a non-ionic dense state, with only minimal eugenol released through concentration-gradient-controlled release. The membrane’s barrier function provides basic preservation while maintaining the light-yellow color panel. As storage progresses, TVB-N elevates local pH to near or above L100’s pKa, triggering deprotonation of L100 and network pore opening, which accelerates eugenol release for enhanced antibacterial and antioxidant effects. During this process, the film transitions from light yellow to orange–yellow, allowing consumers to rapidly assess food quality through the color panel. Brands can leverage this feature to reduce losses, minimize claims, and enhance premium pricing, enabling differentiated competition and quality assurance in the high-end seafood market.

## 3. Conclusions

This chapter employs coaxial electrospinning technology to successfully prepare a core–shell-structured nanofiber membrane for fresh shrimp preservation, where pH-sensitive polymer L100 serves as the shell, and eugenol (EG) as the core. Through systematic characterization of the microstructure, physicochemical properties, and release kinetics of the fiber membrane, the formation of the core–shell structure was confirmed, and its release behavior exhibited significant pH responsiveness under different pH conditions. At a pH of 6.25, the release data best fitted a biphasic kinetic model (R^2^ = 0.9952). Antibacterial experiments demonstrated that the fiber membrane showed notable inhibitory effects against *Escherichia coli* and *Staphylococcus aureus*. In shrimp meat preservation experiments, the L100@EG fiber membrane achieved “self-sensing and self-regulating” coordinated release in response to pH changes during storage, extending the shelf life of fresh shrimp by approximately 6–7 days at 4 °C. However, this study still faces several challenges and limitations. Firstly, regarding the release mechanism, although the biphasic kinetic model demonstrates high fit, the actual release behavior of the active ingredient in complex food matrices may be influenced by factors such as protein degradation products and lipid oxidation, and further optimization is required to achieve precise alignment between the release kinetics and microbial growth curves. Secondly, in terms of material safety, while eugenol as a natural active ingredient exhibits good biocompatibility, its stability during long-term storage and potential impact on the sensory quality of shrimp meat (e.g., odor and color) require systematic evaluation. Additionally, in large-scale production, coaxial electrospinning technology currently faces issues such as low production efficiency and stringent process parameter control requirements, with batch consistency and long-term storage stability of the core–shell structure still needing improvement.

Despite these limitations, the packaging demonstrates significant commercial potential. Its integrated pH sensing, on-demand release, and visual alert functions precisely meet the premium seafood industry’s demands for “smart preservation” and “quality visualization,” establishing a differentiated competitive edge. With the maturation of electrospinning technology, production efficiency is expected to improve, paving the way for large-scale manufacturing. Combining technological sophistication with luxurious aesthetics, this packaging achieves integrated sensing, controlled-release mechanisms, and visual alerts. Through formula optimization, cost reduction, and enhanced safety evaluations, this smart packaging system is poised for commercialization in the premium seafood preservation sector.

## 4. Materials and Methods

### 4.1. Materials and Reagents

Fresh shrimps were purchased from the local fresh market (Tianjin, China). Eudragit L100 was purchased from McLean Biochemical Technology Co., Ltd. (Shanghai, China); TEMPO was purchased from Guangdong Wengjiang Chemical Reagents Co., Ltd. (Shaoguan, China); MCC was purchased from Shanghai Yuan Ye Biotechnology Co., Ltd. (Shanghai, China); CTAC was purchased from Tianjin Kemio Chemical Reagents Co., Ltd. (Tianjin, China); NaBr was purchased from Tianjin Kemio Chemical Reagents Co., Ltd. (Tianjin, China); NaHCO_3_ was purchased from Tianjin Kemio Chemical Reagents Co., Ltd. (Tianjin, China); NaClO was purchased from Tianjin Tianli Chemical Reagents Co., Ltd. (Tianjin, China); DMSO was purchased from Tianjin Guangfu Fine Chemical Research Institute, (Tianjin, China); HCI was purchased from Tianjin Jiangtian Chemical Technology Co., Ltd. (Tianjin, China); Rhizoma curcumae longae was purchased from the local fresh market (Tianjin, China). TG was purchased from Zhejiang Yino Biotechnology Co., Ltd. (Shanghai, China); TiO_2_ was purchased from Shanghai Linen Technology Development Co., Ltd. (Shanghai, China); CaCl_2_ was purchased from Tianjin Jiangtian Chemical Technology Co., Ltd. (Tianjin, China); Gly was purchased from Tianjin Jiangtian Chemical Technology Co., Ltd. (Tianjin, China); alcohol was purchased from Tianjin Jiangtian Chemical Technology Co., Ltd. (Tianjin, China); agal-agal was purchased from Tianjin Jiangtian Chemical Technology Co., Ltd. (Tianjin, China); PBS phosphate buffer was purchased from Aladdin Reagent Co., Ltd. (Shanghai, China); Mg(NO_3_)_2_ was purchased from Aladdin Reagent Co., Ltd. (Shanghai, China); glutaraldehyde was purchased from Aladdin Reagent Co., Ltd. (Shanghai, China); Colibacillus was purchased from Qingdao Haibo Biotechnology Co., Ltd. (Shanghai, China); DPPH was purchased from Aladdin Reagent Co., Ltd. (Shanghai, China); GG was purchased from Shandong Keyuan Biochemical Co., Ltd. (Laizhou, China); aqueous ammonia was purchased from Aladdin Reagent Co., Ltd. (Shanghai, China); Brazil wax was purchased from Shanghai McLean Biochemical Technology Co., Ltd. (Shanghai, China).

### 4.2. Instruments and Equipment

The following experimental instruments were used: Ultrasonic cell pulverizer (LP-XP3000 model, Wuxi LaiPu Instrument Equipment Co., Ltd.); Table model high speed centrifuge (TG16-WS Hunan Xiangli Scientific Instruments Co., Ltd., Wuhan, China); Vacuum drying oven (DZF-6020 Shanghai Boxun Industrial Co., Ltd., Shanghai, China); Magnetic heating mixer (WH220-HT German company, Germany); Scanning electron microscope (HITACHI-S4800 Hitachi Corporation of Japan, Japan); Fourier transform infrared spectrometer (Nicolet-iS50 Thermo Fisher Scientific D8 Discover German company Brook, Shanghai, China); Intelligent electronic tensile testing machine (XLW-PC Jinan Lan Guang Electromechanical Technology Co., Ltd., Jinan, China); Constant Temperature Incubator (LHS-50CH Shanghai Yiheng Scientific Instruments Co., Ltd., Shanghai, China) Water Drop Contact Angle Analyzer Measurement (JC2000DM Shanghai Zhongchen Digital Technology Equipment Co., Ltd., Shanghai, China); Vacuum rotary evaporator (ATR-001 Huachen Instruments); Electronic balance (ES-E120A Tianjin Demeng Technology Co., Ltd., Tianjin, China); UV–visible spectrophotometer (LH-S700 Beijing Lianhua Yongxing Technology Development Co., Ltd., Beijing, China); Portable color difference meter (CM2300d Konica Minolta Co., Ltd.); Electrospinning machine (JDF05 Changsha Nanyi Instrument Technology Co., Ltd., Changsha, Chia); Capillary pore size analyzer (Porolux 1000 Belgian Prometheum Co., Ltd., Guangzhou, China).

### 4.3. Experimental Methods

#### 4.3.1. Preparation of pH-Controlled Sustained-Release Layer

**Figure 16:**
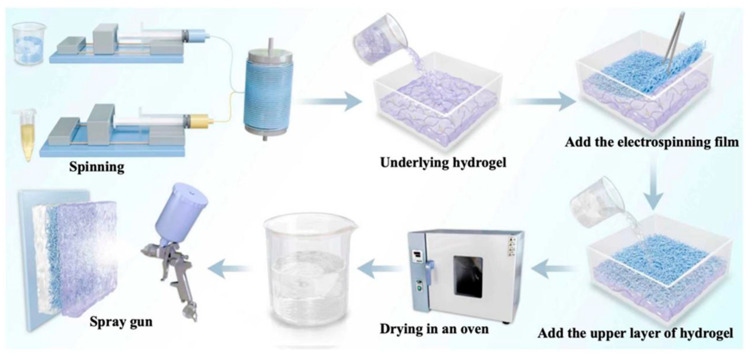
Schematic diagram of the preparation process for pH auto-controlled sustained-release sensor.

**Figure 17:**
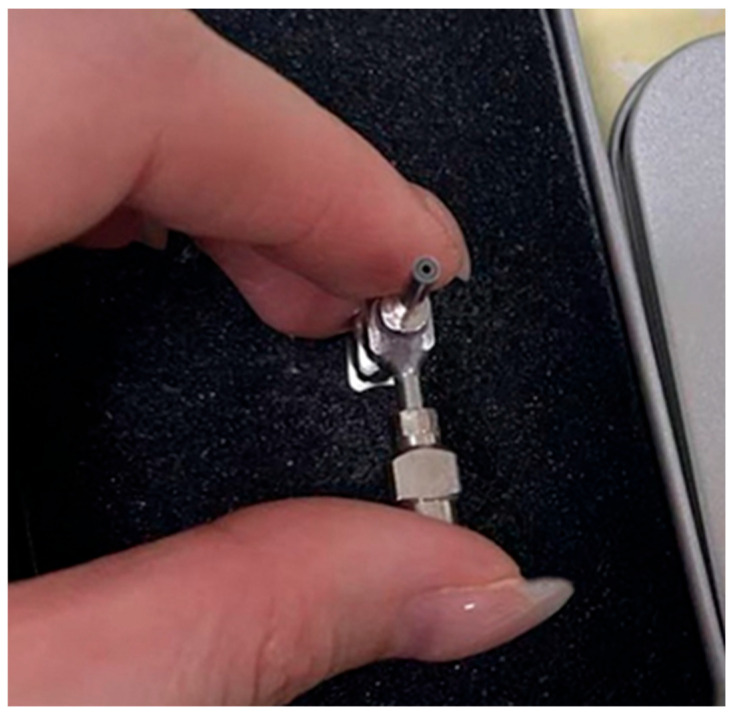
Coaxial needle.

A 15% (*w*/*v*) solution of L100 and citric acid (30:1) was dissolved in 20 mL of ethanol and DMF and then stirred for 3 h. Then, 0%, 2%, 4%, 6%, and 8% (*w*/*w*, L100) of clove essential oil was diluted into the ethanol solution and uniformly stirred to obtain the core-layer solution. The shell-layer solution and core-layer solution were separately loaded into 5 mL syringes, which were mounted on both sides of a coaxial device, as shown in Figure 16, Step 1. The nozzles were selected to match the concentric rings of G21# and G18#, respectively. The inner ring of the concentric ring was connected to the syringe containing the core-layer solution, while the shell-layer syringe was connected to the cross-shaped outer sheath via a flexible tube(Figure 17). The flow rate of the core-layer solution was set to 0.1 mL/h, and the flow rate of the shell layer was set to 1.2 mL/h. The prepared nanofiber membranes were labeled as L100@EG0, L100@EG2, L100@EG4, L100@EG6, and L100@EG8.

#### 4.3.2. Preparation of pH Auto-Controlled Sustained-Release Sensor

First, 2 g of GG was dissolved in 100 mL of water at 98 °C and stirred for 2 h. Then, 5 mL of 8 mg/mL CaCl_2_ solution was added for ion cross-linking. After cooling the solution to 45 °C, 6% (*w*/*w*, GG) CQTCN was added (the surface of CNC was modified by TEMPO oxidation to prepare oxidized cellulose nanocrystals (TCN)). Quaternization modification of TCN was performed using cetyltrimethylammonium bromide (CTAC) to obtain hydrophobic cellulose nanocrystals (QTCN). Cur was loaded into QTCN via physical adsorption to prepare CQTCN, and the mixture was stirred for 30 min. The mixed solution was degassed by ultrasonication and poured into a clean glass Petri dish with a diameter of 53 mm. After cooling at room temperature, a hydrogel substrate was formed, yielding the GG/CQTCN hydrogel. L100@EG pH-controlled release nanofilm with different proportions of eugenol essential oil was placed on the GG/CQTCN hydrogel. Then, 2 g of TG powder added to 100 mL of water at 80 °C and stirred until dissolved. TiO_2_ nanoparticles with a mass concentration of 1% TG were ultrasonically dispersed in 2 mL of water, and the TG solution was added dropwise. Subsequently, ultrasonic stirring was performed for treatment. Finally, 30% Gly (*w*/*w*, TG) was added and stirred for 30 min. When the solution cooled to 50 °C, the TG-TiO_2_ solution was poured onto the prepared GG/CQTCN hydrogel. After cooling, the pH self-regulating slow-release sensor was obtained, which was then dried into a film in a 50 °C vacuum drying oven and carefully peeled off. The film was stabilized for 24 h under a relative humidity of 43%.

The Brazilian palm wax was dissolved in ethanol at 35 °C and stirred until fully dissolved. An appropriate amount of glycerol and citric acid aqueous solution was then added, and the mixture was stirred at room temperature for 120 min. After defoaming, the mixture was transferred to a spray gun and sprayed onto the surface of the pH self-regulating slow-release sensor. The spray gun was set to a pressure of 0.12 MPa and a receiving distance of 20 cm. Samples were labeled according to the proportion of clove essential oil, as follows: TG-GG/L100@EG0, TG-GG/L100@EG2, TG-GG/L100@EG4, TG-GG/L100@EG6, and TG-GG/L100@EG8. The preparation process is illustrated in Figure 17.

#### 4.3.3. Microstructural Characterization of pH Sensors

- Scanning Electron Microscopy (SEM) Observation

The sample was fixed on the sample stage with conductive adhesive; then, the sample was sprayed with gold, and the microstructure of the powder was observed using a scanning electron microscope (SEM) with an acceleration voltage of 15 kV.

- Infrared Spectroscopy (FTIR)

Fourier transform infrared spectroscopy (FTIR) was used to analyze L100 powder, eugenol, and L100@EG fiber membrane. A small amount of each sample was placed on the surface of a diamond crystal attached to an ATR, scanning was performed within the wavenumber range of 4000–525 cm^−1^ with a resolution of 4 cm^−1^, and 32 accumulative scans were conducted.

#### 4.3.4. X-Ray Diffraction Energy-Dispersive Spectroscopy (XRD)

Set the Cukα radiation parameters as follows: tube voltage of 40 kV, tube current of 30 mA, and scanning speed of 5°/min. The cellulose crystallinity was calculated using the Segal method. The calculation formula is

(1) $$CI=(1-I_{am}/I_{002})\times 100\%\;\;$$

where CI is the degree of crystallinity (%), $I_{am}$ is the intensity of the 2θ = 18° diffraction peak and $I_{002}$ is the intensity of the 2θ = 22.6° diffraction peak.

#### 4.3.5. Mechanical Property Measurement

Mechanical properties of L100 nanofiber membrane were tested using a texture analyzer. Prior to testing, the membrane samples were cut into strip specimens measuring 20 mm × 60 mm. The initial clamping distance was set at 40 mm, with a tensile rate of 0.6 mm/s. Stress–strain curves were recorded and plotted.

#### 4.3.6. Water Contact Angle Test

A 20 mm × 20 mm film sample was fixed on a horizontal sample stage. The syringe needle was adjusted to be perpendicular to the center of the film, approximately 1 cm above it, and 5 μL of deionized water was slowly added. The morphology of the water droplet was recorded using a high-speed camera for a 10 s dynamic change. Each sample group was measured three times, and the results were averaged.

#### 4.3.7. Determination of Loading Rate (LC), Encapsulation Efficiency (EE), and Porosity of pH-Controlled Release Fibrous Membranes

Sage essential oil readily volatilizes from nanofiber membranes, making it essential to evaluate the liquid chromatography (LC), enthalpy of absorption (EE), and porosity of L100 nanofiber membranes. A 10 mg sample of L100 nanofiber membrane was immersed in 30 mL of 95% ethanol solution and stirred for 72 h. The solution was diluted to a specific ratio, and its absorbance was measured. The absorbance value must fall within the standard curve range for sage essential oil before calculating the concentration of the essential oil. The formulas for calculating EE and LC are as follows [7]:

(2) $$EE\;(\%)\;=\;\frac{m_{a}}{m_{t}}\;\times \;100\%$$

(3) $$LC\;(\%)=\frac{m_{a}}{m_{c}}\;\times \;100\%$$

In the formula: $m_{a}$ denotes the actual mass of clove essential oil calculated from the standard curve, $m_{t}$ represents the theoretically calculated mass of clove essential oil, and $m_{c}$ indicates the total mass of the fibrous membrane.

Porosity is determined by the ratio of apparent volume to actual volume. First, a fixed-shape nanofiber membrane is taken, and its apparent volume (Va) is calculated based on the membrane’s area and thickness. The actual volume (Vg) is then calculated using the mass of the fiber membrane, the density of L100 polymer (0.55 g/cm^3^), citric acid density (1.54 g/cm^3^), and the density of eugenol (1.03 g/cm^3^). The formula for calculating porosity (P) is as follows [29,30]:

(4) $$P\;(\%)\;=\;(1\;-\;\frac{V_{g}}{V_{a}})\;\times \;100\%$$

#### 4.3.8. Determination of Antioxidant and Antibacterial Properties

*Escherichia coli* (*E. coli*, ATCC-25922TM) and *Staphylococcus aureus* (*S. aureus*, ATCC-25923TM) were inoculated into trypsin-soy broth (TSB) at 37 °C and cultured for 24 h. The bacterial suspensions were then diluted to 1 × 105 CFU/mL using sterile PBS buffer (pH 7.4). According to the American Standard ASTM E2149 [31], an appropriate amount of sterile PBS (pH 7.4) was added to sterile Erlenmeyer flasks, followed by inoculation with sterilized samples and the diluted bacterial suspensions. The cultures were incubated at 37 °C on a 110 rpm shaker. After incubation, the culture medium was gradually diluted to a final concentration of approximately 1 × 103 CFU/mL. Two aliquots of 200 μL each of the diluted *E. coli* or *S. aureus* suspensions were evenly spread on soybean peptone agar (TSA) plates and incubated at 37 °C for 24 h. The bactericidal rate was calculated by counting the number of colonies on three parallel plates.

Antibacterial efficiency (%) = (A − B)/A × 100% (5)

A and B represent the number of colony-forming units (CFU) for the control and sample, respectively.

The antioxidant capacity of membrane materials was evaluated based on their ability to scavenge DPPH radicals. A 5.0 mg DPPH powder was weighed, dissolved in ethanol, and diluted to 100 mL. One milliliter of the prepared membrane solution was accurately pipetted, followed by the addition of 3 mL of a 50 mg/L DPPH solution. After thorough mixing, the mixture was incubated under dark conditions for 15 min. Absorbance at 517 nm was measured, with a blank control using the same volume of solvent. The DPPH radical scavenging rate was calculated as shown in Equation (6).

(6) $$I=\left({AbS}_{0}-{AbS}_{extractive}\right)\;/\;{AbS}_{0}\times 100\%$$

In the formula, ${AbS}_{0}\;$ is the absorbance value when using solvent as blank, while ${AbS}_{extractive}\;$is the absorbance value of the antioxidant in the reaction system.

#### 4.3.9. Cytotoxicity Testing of Sustained-Release Fibrous Membrane

Take a certain mass of L100@EG fibrous membrane for sterilization, dissolve it in cell culture medium, and prepare five different concentrations (3.125, 6.25, 12.5, 25, 50 μg/mL). By cell counting, the cell concentration was adjusted to 1 × 105 cells/mL, and inoculated into a 96-well plate, with approximately 104 cells in each well. A blank group (with only fresh culture medium added) was also set up for control. After incubation in a 37 °C, 5% CO_2_ incubator for 24 h, the 96-well plate was supplemented with the prepared culture medium containing different concentrations of the fiber membrane samples. The plate was then further cultured in a 37 °C, 5% CO_2_ incubator for another 24 h. In the dark, 90 μL of fresh culture medium and 10 μL of CCK8 solution were added to each well. After incubation in the dark in a 37 °C, 5% CO_2_ incubator for 2 h, the absorbance (OD value) at 490 nm was measured using an enzyme-linked spectrophotometer.

#### 4.3.10. Study on the Release Mechanism of pH-Controlled Sustained-Release Layer

Release behavior serves as a key indicator for evaluating the successful construction of pH-controlled release active packaging. To assess the pH-responsive characteristics of the fiber membrane, the in vitro release rates of eugenol were measured in buffer solutions with pH values of 5.50, 5.75, 6.00, and 6.25. The specific procedure involved weighing 100 mg of the nanofiber membrane and placing it into beakers containing 30 mL of buffer solutions with different pH values. The release experiment was conducted under oscillation conditions at 180 rpm. Samples of 0.1 mL of the released solution were periodically collected, diluted by adding 3 mL of the corresponding pH buffer solution, and the absorbance was measured at a wavelength of 287 nm. The concentration and cumulative release rate were calculated based on these measurements. Furthermore, six classical release kinetics models were employed to fit the release curves, aiming to elucidate the release mechanism.

Zero-order model: $\frac{M_{t}}{M_{\infty }}\;=\;kt$; first-order model: $\ln(1\;-\frac{M_{t}}{M_{\infty }})\;=\;-\mathrm{kt}$; Ritger–Peppas model: $\frac{M_{t}}{M_{\infty }}\;=\;kt^{n}$; Higuchi model: $\frac{M_{t}}{M_{\infty }}\;=\;kt^{0.5}$; Hixson–Crowell model: $(1-\frac{M_{t}}{M_{\infty }}{)}^{1/3}\;=\;-kt$; and two-phase kinetic model: ($M_{t}/M_{\propto }=Q_{0}-A\times e^{-\alpha t}-B\times e^{-\beta t}$) [32].

#### 4.3.11. Application of pH Sensor in Freshness Monitoring of Fresh Shrimp

Fresh shrimp purchased on the same day were placed in a 350 mL preservation container. The sensing gas was affixed to the inner surface of the container lid, with the hydrophobic layer facing the shrimp and fish meat. After sealing, the container was stored at −4 °C. Daily measurements of the film color parameters were performed using the CM2300d portable colorimeter, while visual morphological changes of the film were simultaneously captured by a camera [33]. Concurrently, the pH value and volatile basic nitrogen (TVB-N) content of the fish meat were determined in accordance with the standards GB 5009.237-2016 and GB 5009.228-2016 [34,35].

## Figures and Tables

**Table 1:** Material costs for preparing pH-sensing composite membranes with a side length of 20 mm.

Material Name	Potency	Weight	Price
L100	99%	1000 g	15
TG	100%	1000 g	45
GG	100%	1000 g	94
MCC	100%	1000 g	16
Cur	98%	1000 g	400
EG	99%	1000 g	797
TiO_2_	100%	1000 g	12
CTAC	100%	1000 g	130
PVDF	100%	1000 g	40

## Data Availability

No new data were created or analyzed in this study. Data sharing is not applicable to this article.
